# Evaluating positional accuracy using megavoltage cone-beam computed tomography for IMRT with head-and-neck cancer

**DOI:** 10.1093/jrr/rrt143

**Published:** 2014-01-20

**Authors:** Kana Motegi, Ryosuke Kohno, Takashi Ueda, Toshiyuki Shibuya, Takaki Ariji, Mitsuhiko Kawashima, Tetsuo Akimoto

**Affiliations:** National Cancer Center Hospital East, 6-5-1 Kashiwanoha, Kashiwa, Chiba, 277-8577, Japan

**Keywords:** positional accuracy, body mass index, head and neck, IMRT, MV-CBCT

## Abstract

Accurate dose delivery is essential for the success of intensity-modulated radiation therapy (IMRT) for patients with head-and-neck (HN) cancer. Reproducibility of IMRT dose delivery to HN regions can be critically influenced by treatment-related changes in body contours. Moreover, some set-up margins may not be adaptable to positional uncertainties of HN structures at every treatment. To obtain evidence for appropriate set-up margins in various head and neck areas, we prospectively evaluated positional deviation (*δ* values) of four bony landmarks (i.e. the clivus and occipital protuberance for the head region, and the mental protuberance and C5 for the neck region) using megavoltage cone-beam computed tomography during a treatment course. Over 800 *δ* values were analyzed in each translational direction. Positional uncertainties for HN cancer patients undergoing IMRT were evaluated relative to the body mass index. Low positional accuracy was observed for the neck region compared with the head region. For the head region, most of the *δ* was distributed within ±5 mm, and use of the current set-up margin was appropriate. However, the *δ* values for the neck region were within ±8 mm. Especially for overweight patients, a few millimeters needed to be added to give an adequate set-up margin. For accurate dose delivery to targets and to avoid excess exposure to normal tissues, we recommend that the positional verification process be performed before every treatment.

## INTRODUCTION

Accurate dose delivery is essential for the success of intensity-modulated radiation therapy (IMRT) in patients with head-and-neck (HN) cancer, due to the steep dose gradient between the planning target volume (PTV) and the adjacent organs at risk (e.g. spinal cord and parotid glands). Reproducibility of the patient's position during IMRT is critically important. In general, the patient is immobilized with a customized thermoplastic mask and pillows. The body is positioned on a couch by matching external marks on the mask to the isocenter indicated by lasers. Skin marks on the patient's shoulders and chest are used to assist set-up.

Researchers have used various imaging procedures [1], such as orthogonal mega- or kilovoltage (kV) X-ray radiographic imaging [2–4], computed tomography (CT) on rails [5], and 3D cone-beam computed tomography (CBCT) [4, 6, 7], to verify patient positioning during set-up for IMRT. A recent study evaluated the positional accuracy of HN cancer patients using 3D imaging procedures, revealing positional deviations of 3 mm and ≥5 mm in 18.7 and 4.1% of set-ups, respectively, with kV-CBCT, compared with 11.2 and 1.7%, respectively, with 2D kV radiographic imaging [4]. Differences between the procedures were mainly attributed to the relative flexibility and possible rotation of the HN structures. Complex patterns of set-up errors resulting from these complications were also observed when CT on rails, which found a difference of 2–6 mm for the distance between two bony landmarks at the second or sixth cervical vertebra and the palatine process of the maxillary bone [1]. Various magnitudes of set-up errors among multiple regions-of-interest, which were frequently larger than those detected at the isocenter, were observed using kV-CBCT [7]. When 3D imaging procedures were used, geometrical uncertainties caused by the rotation/flexibility of HN structures became apparent. These findings imply that a variety of set-up margins are required for the different portions of the head and neck during IMRT planning.

Reproducibility of HN IMRT delivery could be critically influenced by changes in body contours derived from e.g. malnutrition or loss of postsurgical edema during treatment [8, 9]. In addition to loose fitting of immobilization masks and pillows, such changes may cause unexpected over- or under-IMRT dosing to targets and critical organs, which should be corrected with replanning of IMRT [10–12]. Set-up margins applied for the clinical target volume (CTV) and the risk organs are decided on the basis of clinical experiences and reported values. Thus, these margins may not be adaptable to the geometrical uncertainties of patient positioning during IMRT, such as the rotation/flexibility of HN structures and changes in body contours.

To obtain evidence for appropriate set-up margins for various HN portions, using 3D megavoltage (MV)-CBCT, we prospectively evaluated the positional deviation for four bony landmarks (clivus, occipital protuberance, mental protuberance, and C5) during a course of HN IMRT. Additionally, because obese patients generally have difficulty maintaining their weight during treatment and tend to have low positional reproducibility, positional uncertainties were evaluated relative to the body mass index (BMI).

## MATERIALS AND METHODS

### Patient characteristics and set-up

**Fig. 1.**
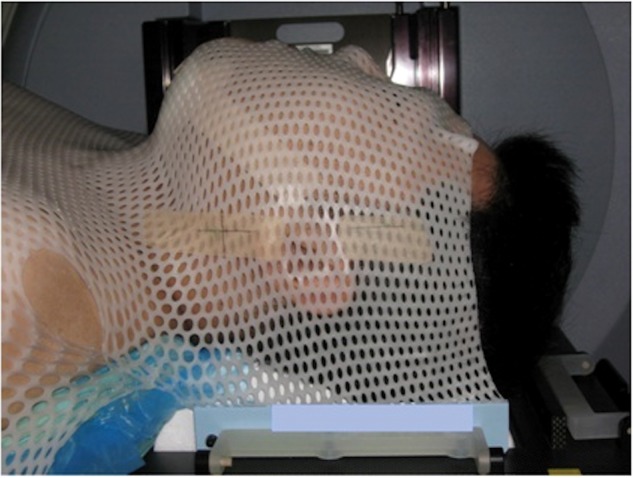
Patient immobilization for HN IMRT.

A total of 67 patients with HN cancer who underwent IMRT were included in this study. The study was approved by our institution's protocol review board, and patients gave their written consent prior to their participation. Characteristics for the patients are listed in Table 1. As defined by the World Health Organization, patients with BMI < 18, 18 ≤ BMI < 25, and 25 ≤ BMI were classified as underweight, normal weight and overweight, respectively. For set-up, all patients were immobilized in a supine position, with thermoplastic fixation masks and customized vacuum pillows extended to the shoulders from the back of the head (Fig. 1). Fixation devices were attached to the treatment couch by an index bar.

**Table 1.** Patient characteristics

Characteristics	
Sex (*n*)	
Male	51
Female	16
Total	67
Age (y)	
Median (Min.–Max.)	59 (18–82)
BMI classification (*n*)	
Underweight (BMI < 18)	10
Normal weight (18 ≤ BMI < 25)	37
Overweight (25 ≤ BMI)	12
Unknown	8
Irradiated site (*n*)	
Nasopharynx	13
Oropharynx	15
Hypopharynx	7
Parotid	3
Paranasal sinus	6
Oral cavity	15
Neck	6
Unknown	2

### Volume acquisition

Once patients were positioned on a treatment couch using their personal masks and vacuum pillows, they were scanned by MV-CBCT mounted on an Oncor linear accelerator (Siemens Medical Solutions, Concord, CA). An amorphous-silicon flat panel detector with an active detector area of 41 cm × 41 cm and a spatial resolution of 1024 × 1024 pixels was used for volumetric acquisition of the MV-CBCT. The voxel size of the reconstructed images was 1.07 mm × 1.07 mm × 1 mm. The maximum field of view (FOV) was 27.4 cm × 27.4 cm at a source-to-axis distance of 100 cm. CT images were reconstructed using 200 projections during 200º of gantry rotation. The CBCT image reconstruction process has been described elsewhere [13–15]. Geometrical distortion of the reconstructed images was evaluated using a phantom with 1-cm square grids. Exposure corresponded to 2.1 cGy at a depth of 10 cm in solid water slabs measured with an ionization chamber.

To avoid clinical overloading and excess exposure to patients, volume acquisition was scheduled as follows: continuously during the first 5 d to confirm reproducibility of the isocenter marking on the masks, and once every 5 d thereafter. When a set-up error ≥3mm was detected, another volume acquisition was performed the next day.

### Verification of patient position

**Fig. 2.**
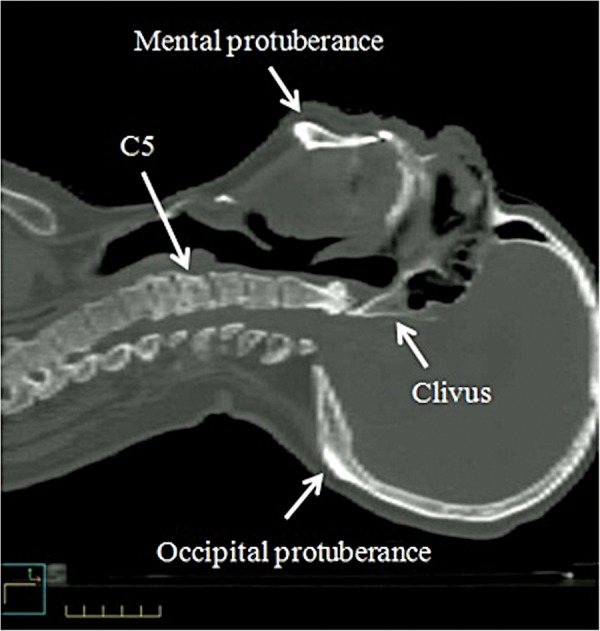
Verification of patient position in HN IMRT. Positional reproducibility was evaluated with four body landmarks: the clivus, the occipital protuberance, the mental protuberance, and C5.

Figure 2 shows the four bony landmarks used for the verification of patient position. The clivus was used for positional verification in the skull because, in many cases, a steep dose gradient is observed at this site to spare the brain stem. The occipital/mental protuberances and C5 were selected to evaluate deviation due to HN flexibility/rotation. For each landmark, discrepancy of the position between treatment and treatment planning (*δ*) was measured by manual registration between MV-CBCT and simulation-CT (Toshiba Medical Systems, Tokyo, Japan) using MVision software (Siemens Medical Solutions, Concord, CA). Simulation-CT had a voxel size of 1 mm × 1 mm × 1 mm. Since the edge of bony structures was detected simply in the CT images compared with the center of bony structures, the edge of bony structures was used in the manual registration. To improve reproducibility of the positional verification, the manual registration was performed changing the contrast of the CT images variously and widely, and prevented the edge of bony structures from being missing on CT images. Therapists were trained in the manual to reduce variations between individuals.

### Statistical analysis

For each landmark, > 800 *δ* values were analyzed in the three translational directions of left–right (LR), craniocaudal (CC), and anteroposterior (AP). The mean and range (minimum to maximum) of *δ* values were obtained. To evaluate positional accuracy for HN IMRT, 1σ of *δ* values were calculated. The statistical analysis was performed for all patients, and patients were categorized by BMI.

## RESULTS

Table 2 shows statistics of the *δ* values for each landmark. Overall, patients tended to shift to the left, caudal and dorsal side within 2 mm. The 1σ values for the clivus and occipital protuberance (range, 1.2–1.7 mm) were less than those for the mental protuberance and C5 (range, 1.5–2.3 mm). Thus, the neck region (mental protuberance and C5) had lower positional accuracy than the head region (clivus and occipital protuberance). The mental protuberance had maximum *δ* and 1σ of 1 cm and 2.8 mm, respectively, which were found in overweight patients.

**Table 2.** Statistical analysis of *δ*^a^

		Clivus			Occipital protuberance			Mental protuberance			C5		
		LR	CC	AP	LR	CC	AP	LR	CC	AP	LR	CC	AP
All patients	Number of *δ* values	867	858	858	824	824	824	813	813	813	831	831	831
	Median (mm)	−1	1	0	−1	0	1	0	1	0	0	1	2
	(Range)	(−4 to 3)	(−4 to 6)	(−3 to 4)	(−6 to 3)	(−7 to 6)	(−4 to 5)	(−4 to 5)	(−7 to 8)	(−8 to 10)	(−8 to 6)	(−6 to 6)	(−7 to 9)
	1σ (mm)	1.2	1.4	1.2	1.4	1.7	1.3	1.5	2.3	1.9	1.9	1.7	2.3
Underweight	Number of *δ* values	123	123	123	123	123	123	112	112	112	112	112	112
	Median (mm)	−1	1	1	−1	1	1	1	1	0	0	1	2
	(Range)	(−3 to 2)	(−3 to 6)	(−1 to 4)	(−5 to 3)	(−4 to 6)	(−2 to 4)	(−3 to 4)	(−2 to 4)	(−2 to 4)	(−5 to 5)	(−2 to 5)	(−2 to 7)
	1σ (mm)	1.3	1.5	0.9	1.4	1.6	1.1	1.6	1.5	1.2	1.7	1.5	1.8
Normal-weight	Number of *δ* values	512	512	512	501	501	501	501	501	501	501	501	501
	Median (mm)	−1	1	0	−1	0	1	0	2	0	0	1	1
	(Range)	(−4 to 2)	(−4 to 5)	(−3 to 4)	(−6 to 3)	(−5 to 5)	(−4 to 5)	(−4 to 5)	(−6 to 7)	(−8 to 6)	(−7 to 4)	(−4 to 6)	(−4 to 8)
	1σ (mm)	1.1	1.4	1.2	1.3	1.7	1.3	1.5	2.2	1.7	1.6	1.7	2.2
Overweight	Number of *δ* values	158	158	158	147	147	147	158	158	158	158	158	158
	Median (mm)	0	0	1	−1	−1	1	1	1	0	−1	0	2
	(Range)	(−4 to 2)	(−4 to 5)	(−3 to 4)	(−4 to 2)	(−7 to 4)	(−3 to 4)	(−4 to 4)	(−7 to 8)	(−4 to 10)	(−8 to 6)	(−6 to 3)	(−4 to 9)
	1σ (mm)	1.2	1.5	1.2	1.4	1.9	1.2	1.5	2.8	2.6	2.3	1.8	2.1

Translational directions are expressed in left–right (LR), craniocaudal (CC), and anteroposterior (AP) directions. ^a^Discrepancy of the position between treatment and treatment planning.

**Fig. 3.**
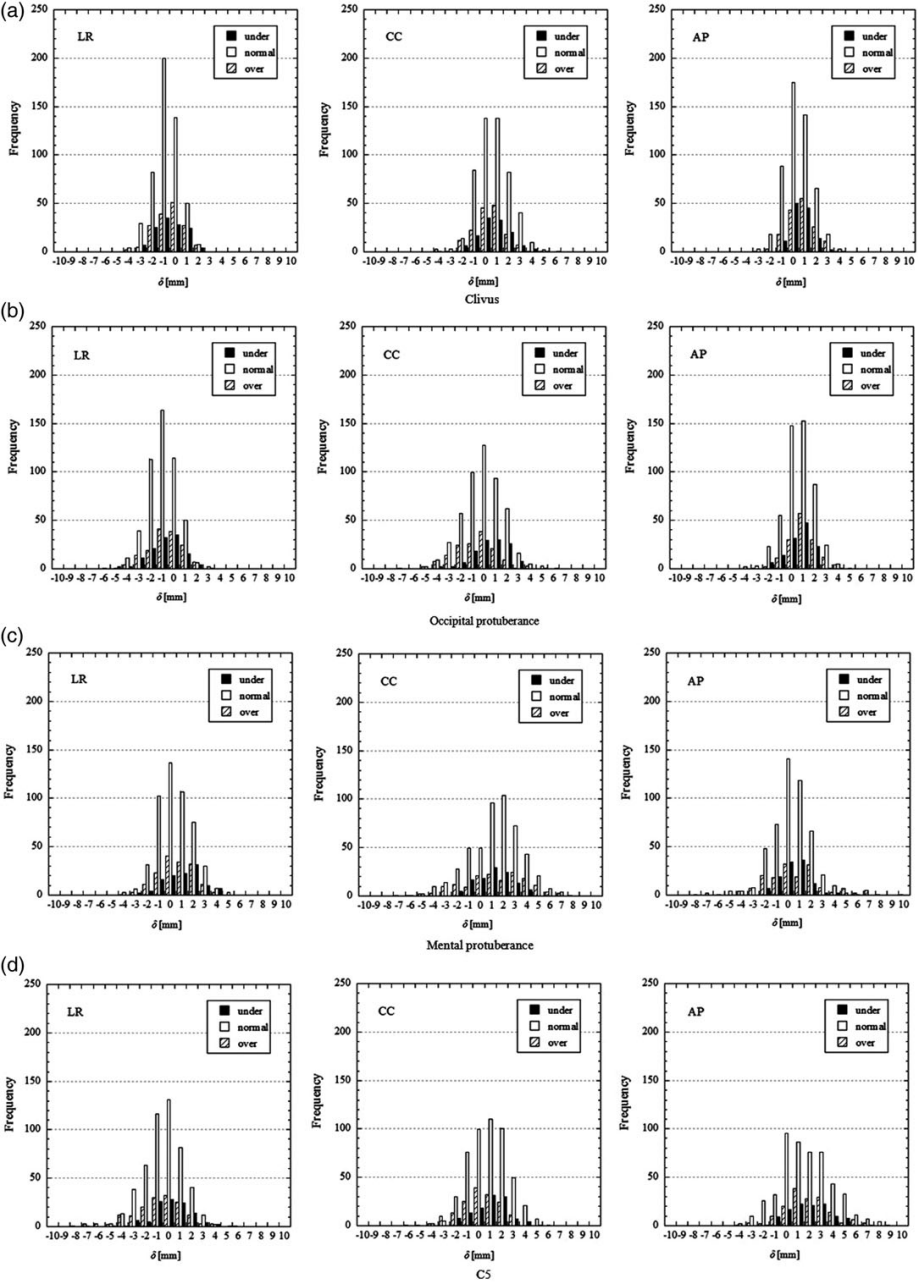
Frequency distribution of positional deviation for four bony landmarks: (**a**) the clivus, (**b**) the occipital protuberance, (**c**) the mental protuberance, and (**d**) C5. Positional reproducibility was evaluated in three translational directions of left–right (LR), craniocaudal (CC) and anteroposterior (AP). Moreover, patients were classified into underweight (under), normal weight (normal), and overweight (over).

To evaluate differences in positional accuracy by BMI, *δ* values were plotted with the frequency distributions (Fig. 3). The positive side of the horizontal axis in Fig. 3 represents the right, caudal and dorsal side of the patients, and the vertical axis represents frequencies. For the clivus (a), occipital protuberance (b), mental protuberance (c), and C5 (d), the *δ* values were distributed in a near-normal distribution. Generally, most of the *δ* values for the clivus and occipital protuberance were distributed within ±5 mm, whereas those for the mental protuberance and C5 were distributed within ±8 mm. Compared with normal weight patients, overweight patients had a wider and more even distribution of *δ* values in the mental protuberance and C5. In underweight patients, the mental protuberance was shifted to the right side, whereas it was left-shifted for normal weight and overweight patients. To evaluate equality of the positional accuracy among the patient groups, an *F*-test was performed on the *δ* values classified by the patient's BMI. The significance level was determined to <0.02 by using a Bonferroni correction for the multiple comparison. In the neck region, (including mental protuberance and C5), the variances of *δ* values were more significantly different among the patient groups than in the head region (including the clivus and the occipital protuberance). Therefore, it was implied that the positional accuracy for the neck region, including the mental protuberance and C5, tended to be affected by the patient's BMI.

In the time trend of *δ* values, patients tended to shift slightly to the right and foot side during a course of treatment. A maximum shift of 0.8 mm to the foot direction was observed for the mental protuberance. No shift along the AP direction was observed in any landmark. Patients maintained constant positional accuracy during a course of treatment.

## DISCUSSION

In this study, lower positional reproducibility was found in the neck region compared with the head region of HN cancer patients, and the patient's BMI affected the positional accuracy of HN IMRT. Overweight patients generally lose fat easily from under the lower jaw, the back of the neck, and the shoulders. The resulting looseness of the fixation mask markedly reduced the reproducibility of patient positioning. It will be very interesting to evaluate the relation between patients' surfaces and positional accuracy among the patient groups using volume data such as CBCT. In addition, it is potentially difficult to make fixation masks that will adjust to rapid differences in shape between the lower neck and upper chest.

In treatment planning for HN IMRT, a set-up margin of 5 mm was applied to any portion of the HN region. Most of the *δ* values for the clivus and occipital protuberance were distributed within ±5 mm. Thus, the current set-up margin for the head region was reasonably adequate. On the other hand, most of the *δ* values for the mental protuberance and C5 were within ±8 mm, suggesting that the set-up margin for the neck region should be expanded by a few millimeters. Furthermore, the frequency of |*δ*| > 5 mm was evaluated (Table 3). In particular, the percentage of |*δ*| > 5 mm out of the number of *δ* values for overweight patients was > 5% in the CC and AP directions for the mental protuberance, and the LR and AP directions for C5.

**Table 3.** Evaluation of the current set-up margin for HN IMRT

	Number of |*δ*^a^| >5 mm (%^b^)											
	Clivus			Occipital protuberance			Mental protuberance			C5		
	LR	CC	AP	LR	CC	AP	LR	CC	AP	LR	CC	AP
All patients	0 (0.0)	1 (0.1)	0 (0.0)	1 (0.1)	2 (0.2)	0 (0.0)	0 (0.0)	23 (2.8)	14 (1.7)	12 (1.4)	2 (0.2)	43 (5.2)
Underweight	0 (0.0)	1 (0.8)	0 (0.0)	0 (0.0)	1 (0.8)	0 (0.0)	0 (0.0)	0 (0.0)	0 (0.0)	0 (0.0)	0 (0.0)	2 (1.8)
Normal weight	0 (0.0)	0 (0.0)	0 (0.0)	1 (0.2)	0 (0.0)	0 (0.0)	0 (0.0)	13 (2.6)	5 (1.0)	1 (0.2)	1 (0.2)	22 (4.4)
Overweight	0 (0.0)	0 (0.0)	0 (0.0)	0 (0.0)	1 (0.7)	0 (0.0)	0 (0.0)	10 (6.3)	9 (5.7)	8 (5.1)	1 (0.6)	10 (6.3)

^a^Discrepancy of the position between treatment and treatment planning. ^b^Percentage of |*δ*| >5 mm in patient groups.

It was reported that the positional deviation of patients decreased the dose delivered to the PTV by 3–14% and caused excess exposure to critical organs [4]. Thus, positional verification before beam delivery is essential for successful HN IMRT. However, many facilities schedule specific days for the positional verification and do not perform such verification at every treatment. Therefore, we strongly suggest that the positional verification process be repeated frequently, preferably before every treatment, to prevent excess radiation exposure to adjacent critical organs by expansion of the PTV. In this context, the use of recent low-exposure 3D imaging devices in image-guided radiation therapy may be very useful.

## CONCLUSION

Deviation of the patient position during a course of treatment was evaluated in HN IMRT. The positional deviation was locally different in the HN regions, with lower positional reproducibility being observed in the neck. Overweight patients had the lowest positional accuracies. An increase in the set-up margin of a few millimeters was required if the CTV and critical organs were located in the neck region. For accurate dose delivery to targets and to spare normal tissues, we recommend repeating the positional verification process often and using image-guided radiation therapy.
